# Planar compact four port MIMO antenna for Ultra Wideband applications

**DOI:** 10.1371/journal.pone.0314193

**Published:** 2024-12-02

**Authors:** Poonam Thanki, Trushit Upadhyaya, Upesh Patel, Vishal Sorathiya, Mohammad Khishe

**Affiliations:** 1 V. T. Patel Department of Electronics and Communication Engineering, Chandubhai S. Patel Institute of Technology, Charotar University of Science and Technology (CHARUSAT), Changa, India; 2 Parul Institute of Engineering and Technology, Parul University, Vadodara, Gujarat, India; 3 Department of Electrical Engineering, Imam Khomeini Naval Science University of Nowshahr, Nowshahr, Iran; 4 Innovation Center for Artificial Intelligence Applications, Yuan Ze University, Taoyuan, Taiwan; 5 Applied Science Research Center, Applied Science Private University, Amman, Jordan; Polytechnique Montreal (University of Montreal) / Microchip Inc, CANADA

## Abstract

This work presents a small four-port multiple-input multiple-output (MIMO) antenna for Ultra Wideband (UWB) applications. Four monopole radiating components make up the suggested antenna. Every monopole is positioned perpendicularly to the components that surround it. This compact antenna, 40 mm × 40 mm, is printed on a single layer substrate (FR4) with a thickness of 1.6 mm and an ε_r_ = 4.4. This antenna features an isolation of less than −14 dB and an impedance bandwidth (S11 < −10 dB) of 2.57–12.20 GHz. The average gain is 4.7 dBi and the envelope correction coefficient (ECC) is less than 0.15. The suggested antenna is a good option for UWB applications because of its Ultra Wide bandwidth and small footprint.

## 1. Introduction

The benefits of planar antennas including their compact size, low profile, easy layout, affordable cost, and easy connection with other high-frequency devices have drawn a lot of interest from both the wireless industry and researchers Peer-to-peer ultrafast communications, short-range communications, and other high data rate current wireless applications are the greatest uses for these antennas [1]. The ability of multiple-input-multiple-output (MIMO) antenna configurations to adapt to the environment of exponentially rising traffic has garnered significant attention recently. While maintaining the same frequency range and transmit power, MIMO technology may significantly reduce the impacts of multipath fading and increase data rate, range, and reliability. Several antenna components are often combined together with minimal mutual coupling between the antenna ports to create a high-performing MIMO system [2–4]. However, adjusting several antenna components within a portable device might be challenging due to their small physical area. The radiating antenna unit cells are subjected to high inter-element coupling due to several closely-operating bands and space constraints, resulting in a degradation of the system performance [5–7]. Numerous investigators have reported seeing several MIMO antennas in the past few years [8–13].

In recent years, a significant amount of research has been done on MIMO antennas as crucial components of UWB MIMO systems. To increase the antennas’ isolation, a few slots are cut into the ground plane in [14]. To achieve great isolation, two inverted C-shaped ground metal strips are positioned in between the two radiating components [15]. In order to increase the isolation among the radiating components and create various resonances, stub of different shape is placed in the defective ground in [16]. The improvement of isolation is accomplished in [17, 18] by incorporating slots and slotted strips into the defective ground plane. The isolation of two antenna elements is enhanced in [19] by the use of very expensive carbon black film. The literatures address many methods for obtaining UWB in MIMO antennas and different applications of MIMO antenna [20–28].

This article presents a UWB MIMO antenna with good isolation and low footprint. A monopole antenna forms up the suggested MIMO antenna. Without the need for further coupling or decoupling mechanisms, good isolation is attained. The impedance bandwidth of the suggested antenna is 2.57–12.20 GHz. Between 2.57 GHz and 12.20 GHz, there is less than −14 dB of mutual coupling.

## 2. Geometry of antenna

### 2.1. Single monopole antenna element

**Fig 1:**
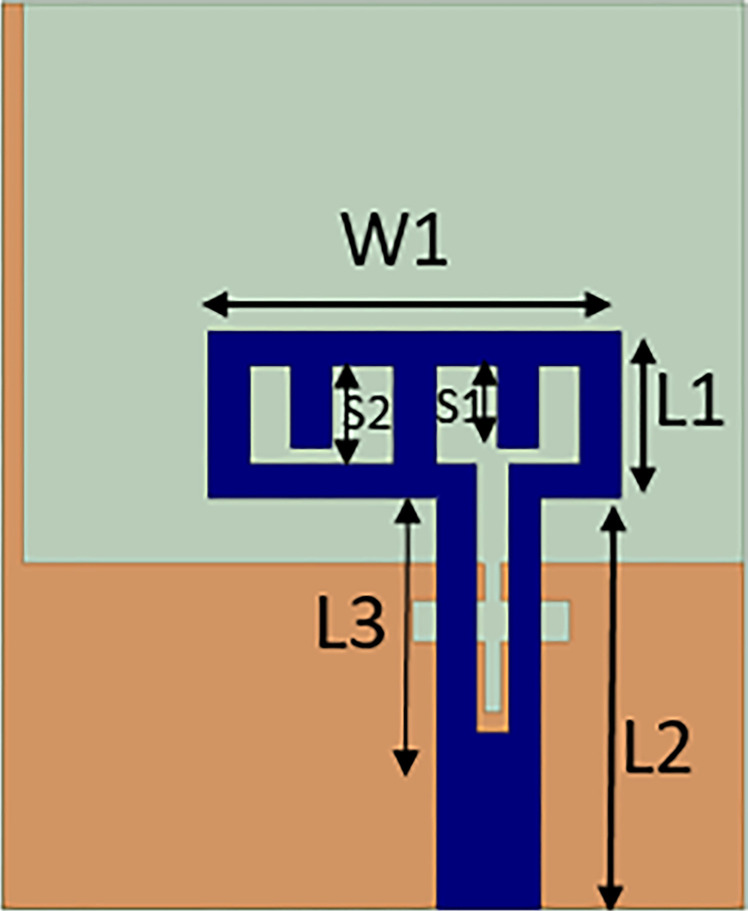
Single monopole antenna design.

The suggested monopole antenna is 22mm x 18mm. The substrate is FR-4, with 1.6 mm thickness, a loss tangent of 0.02, and a dielectric constant of 4.4. The ground plane length is truncated to achieve the desired frequency without altering antenna dimension. With change in length of ground plane, different resonant frequencies can be achieved but it causes impedance mismatch. In the provided antenna design, feeding line width is optimized to match the impedance at desired frequency. Fig 1 shows the antenna design of single monopole. Table 1 shows the optimized parameter values of the designed antenna.

**Table 1:** Optimized parameters of the designed antenna (in mm).

W	L	W1	W2	W3	L1	L2	L3
40	40	10	3.8	0.4	4	10	6.5
L4	L5	L6	L7	S1	S2	S3	
8.38	1	3.6	18	2	2.4	1	

#### 2.1.1. Single element monopole design evolution

**Fig 2:**
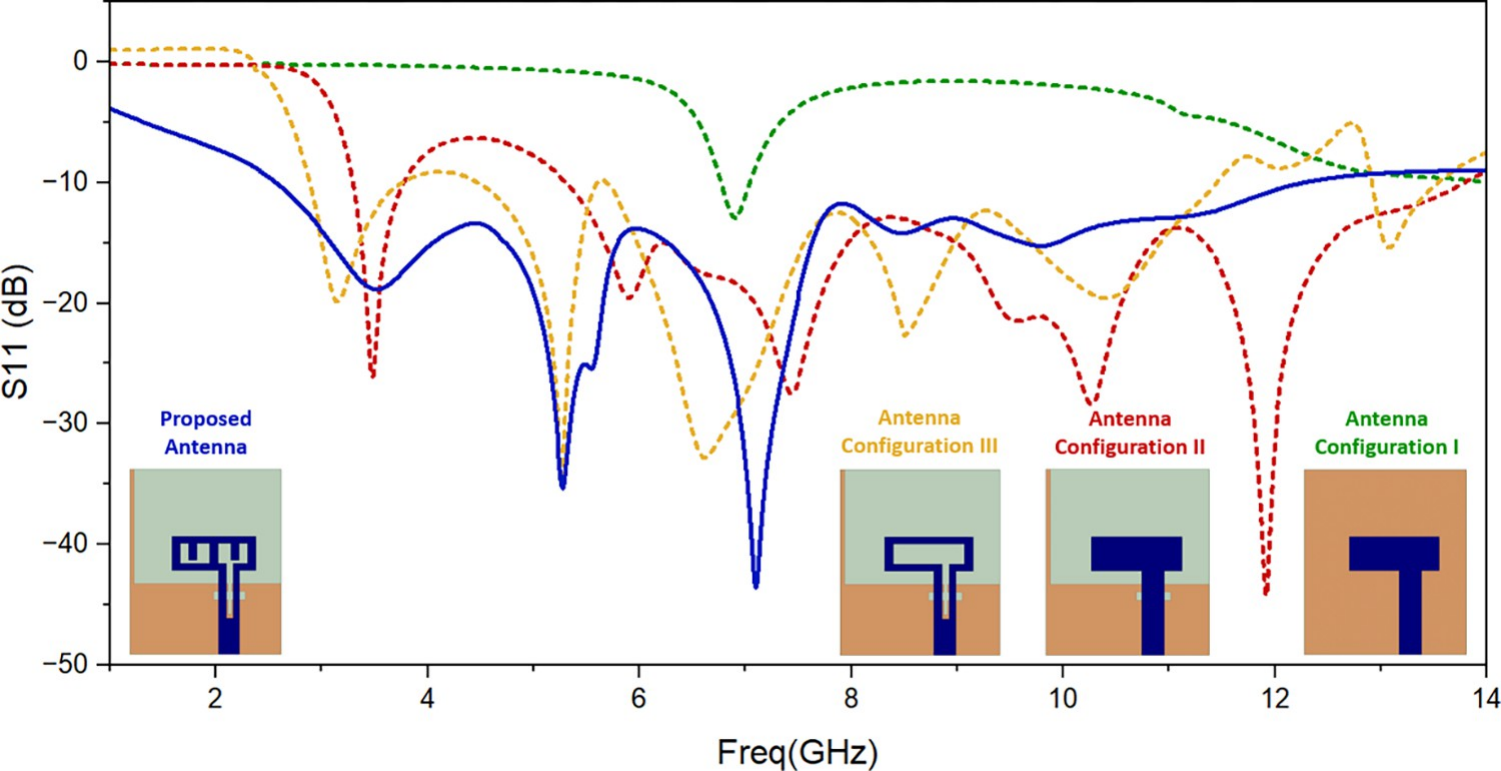
Simulated return loss of the evolution steps.

Fig 2 shows simulated result of return loss for different antenna configurations. Antenna Configuration I illustrates how an antenna with an L-shaped antenna element and less than half of the ground plane is first developed. It is noticed that the antenna resonates at around 7GHz frequency. The ground plane is then refined (Antenna Configuration II) in order to emit for the needed frequency band. Further to obtain required frequency band, slots are created to derive proposed antenna. So it can be seen that slots of different length are responsible for different resonances.

#### 2.1.2. Parametric analysis

**Fig 3:**
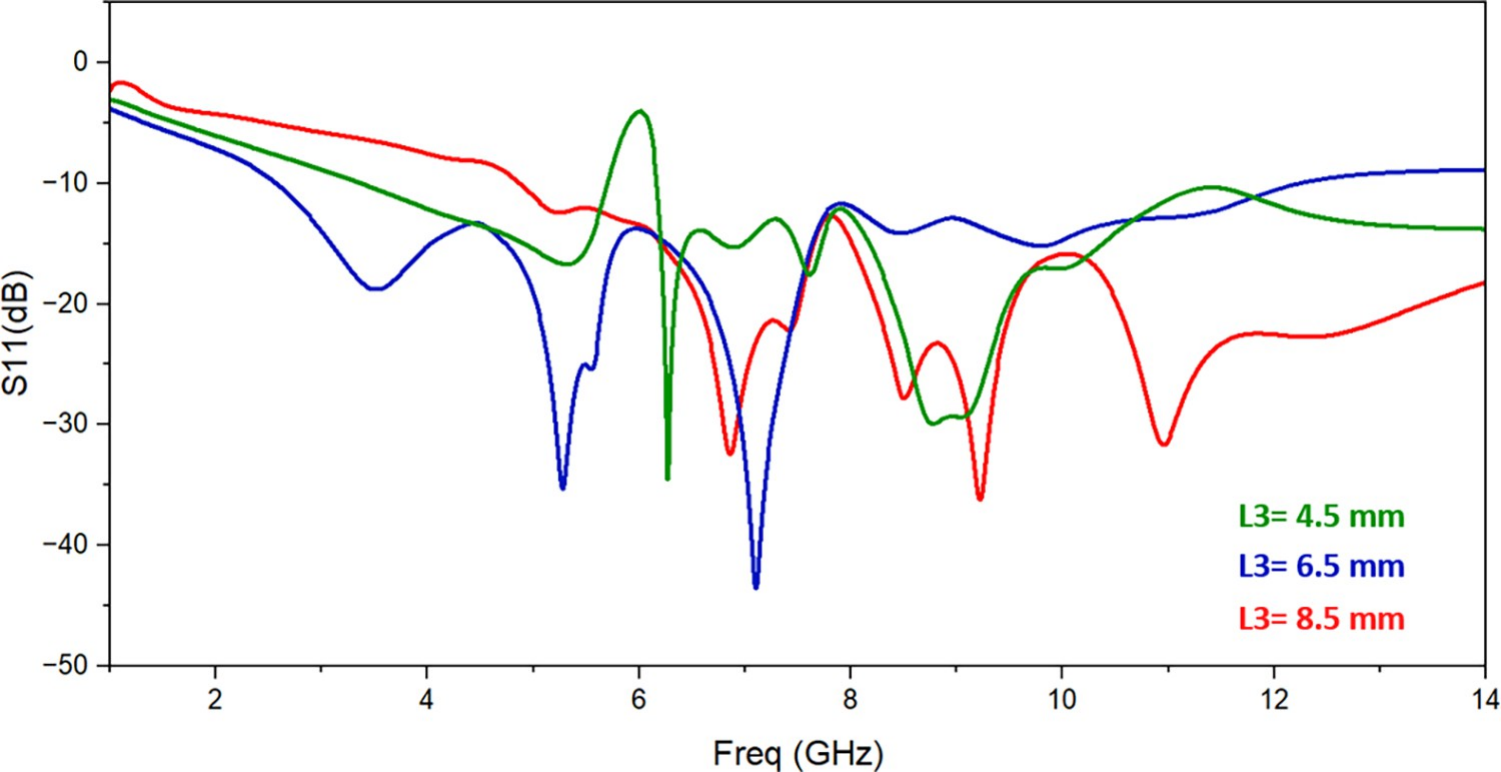
Effect of change in feedline slot length L3.

**Fig 4:**
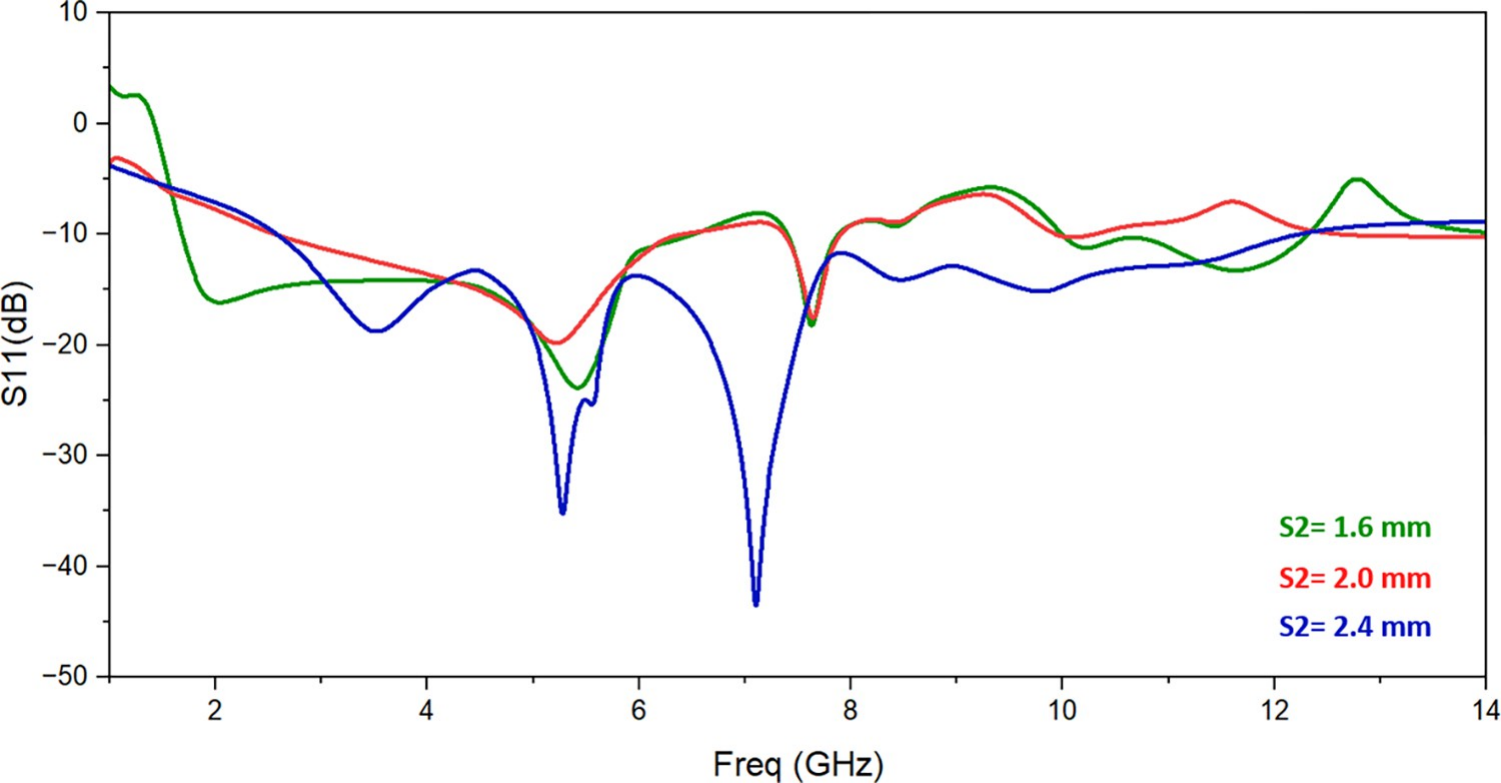
Effect of change in slot length S2.

**Fig 5:**
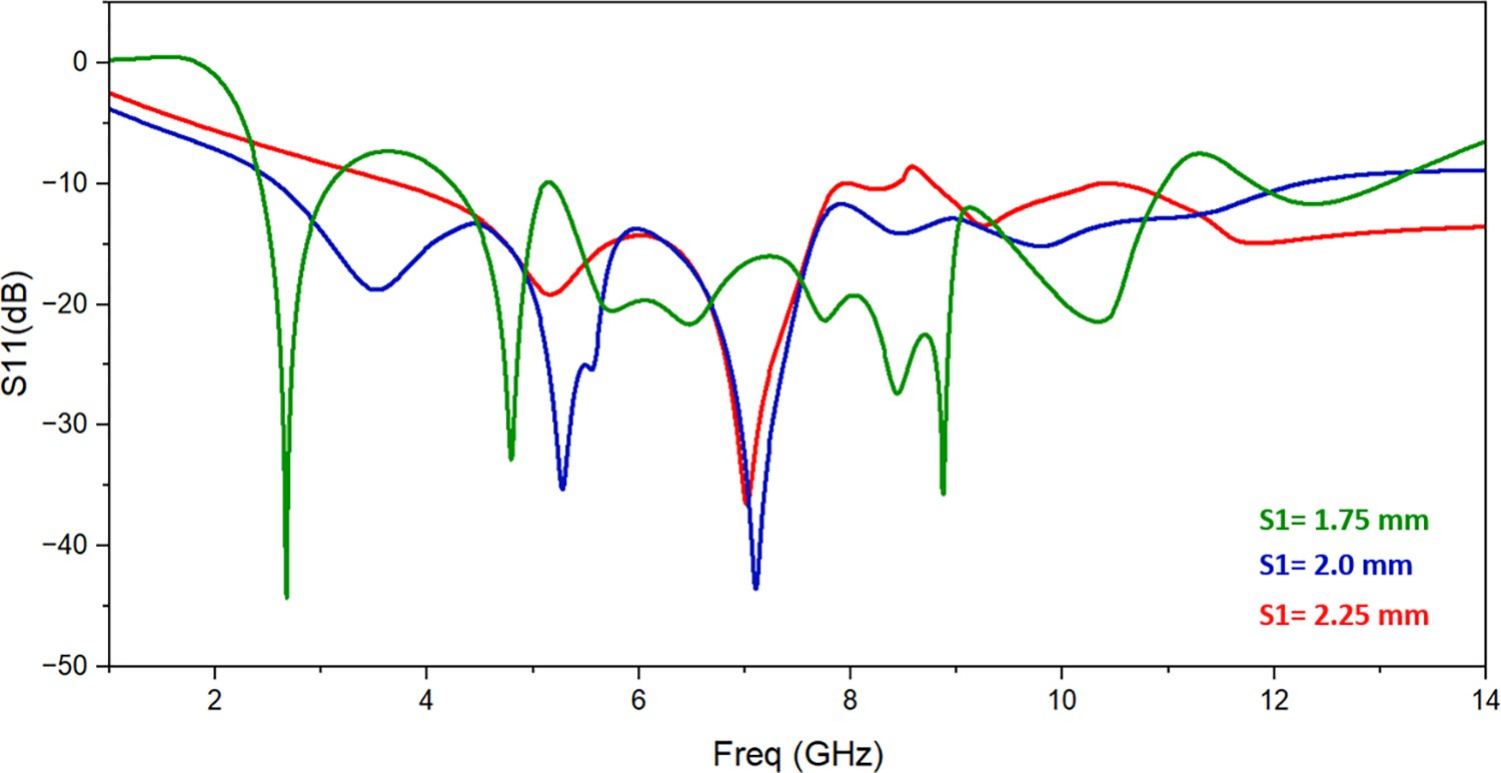
Effect of change in slot length S1.

The single monopole element is subjected to parametric analysis in order to comprehend the change in resonant frequency as a result of various parameters. The variation in L3 changes the operating frequency as shown in Fig 3. The value of L3 is set to 6.5 mm to achieve appropriated frequency range. Similarly, slot S2’s dimensions are altered from 1.6 mm to 2.4 mm while keeping all other parameter values the same in order to comprehend the impacts of employing slot S2. Fig 4 illustrates how the return-loss S11 progressively gets better, spanning the necessary frequency range for S2 = 2.4 mm. The variation of the resonant frequency with the value of S1 is seen in Fig 5. To reach the intended frequency range, S1’s value is therefore adjusted to 2 mm.

### 2.2. Four port MIMO proposed antenna

**Fig 6:**
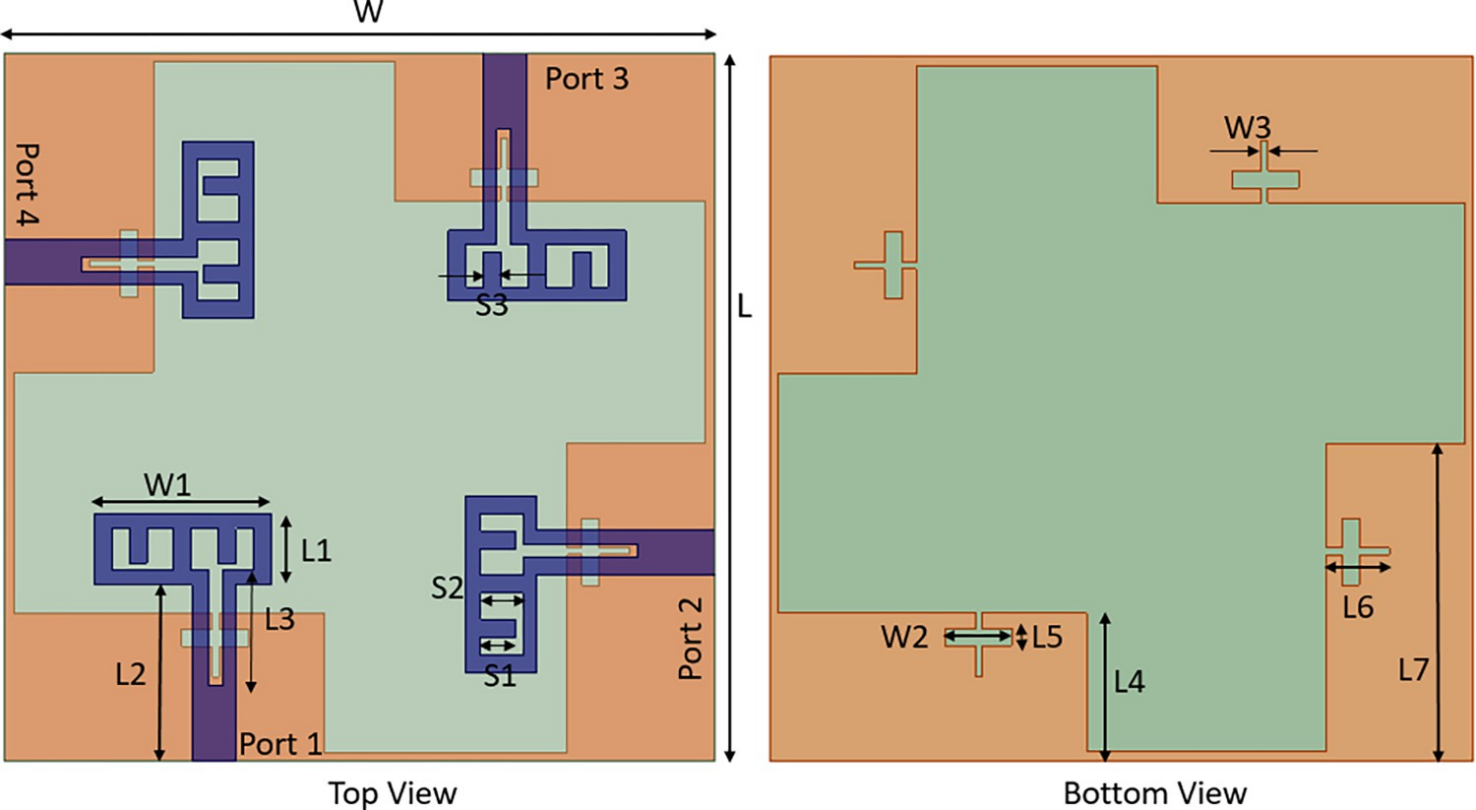
Four port MIMO antenna configuration.

The proposed four port antenna’s 2D layout structure is seen in Fig 6. The antenna measures 40 mm x 40 mm in size. The antenna at port 2 faces the same direction as the antenna at port 4, but it is oriented perpendicularly to the antennas at ports 1 and 3. Additionally, the orientation of the antenna at port 3 is perpendicular to that of the antenna at port 2, port 4, and identical to that of the antenna at port 1 in the opposite direction. Increasing the number of MIMO antenna elements increase the difficulty of decoupling. Therefore, each element of the four-element MIMO antenna is placed orthogonally. The orthogonal placement of antenna elements leads to the polarization mismatch of adjacent antennas, thus improving the isolation degree between antenna elements.

A separate ground plane can enhance port isolation as it has no current coupling. However, since each signal in a practical system should have a common ground plane, this technique is inappropriate [29]. In this investigation, a connected ground design is thus used. Since a very thin 0.5 mm strip line is employed to link all ground planes, it little affects the antenna performance. The width and position of strip line is optimized after several iterations. Optimized parameters of designed antenna are listed in Table 1.

**Fig 7:**
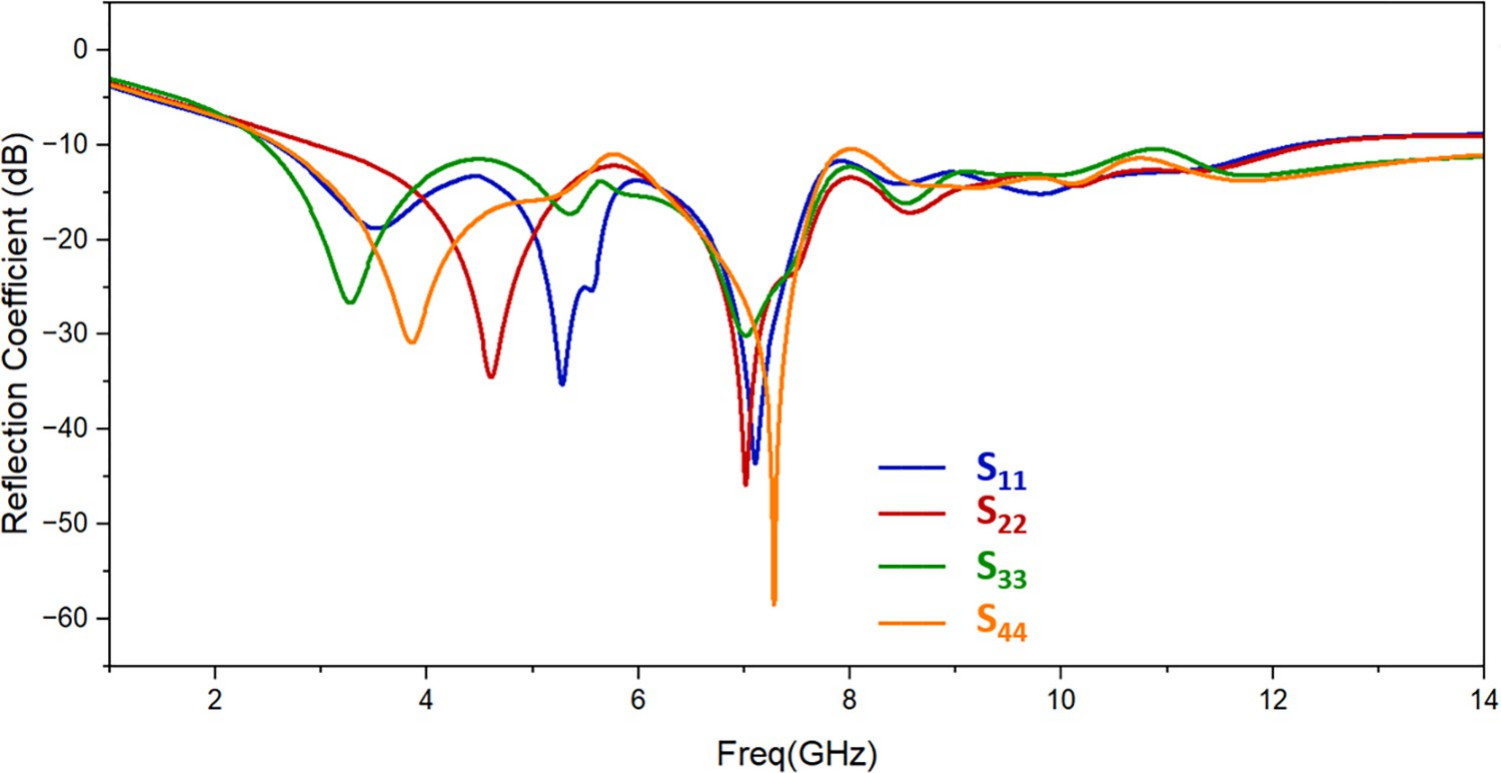
Simulation of the proposed antenna’s return loss.

**Fig 8:**
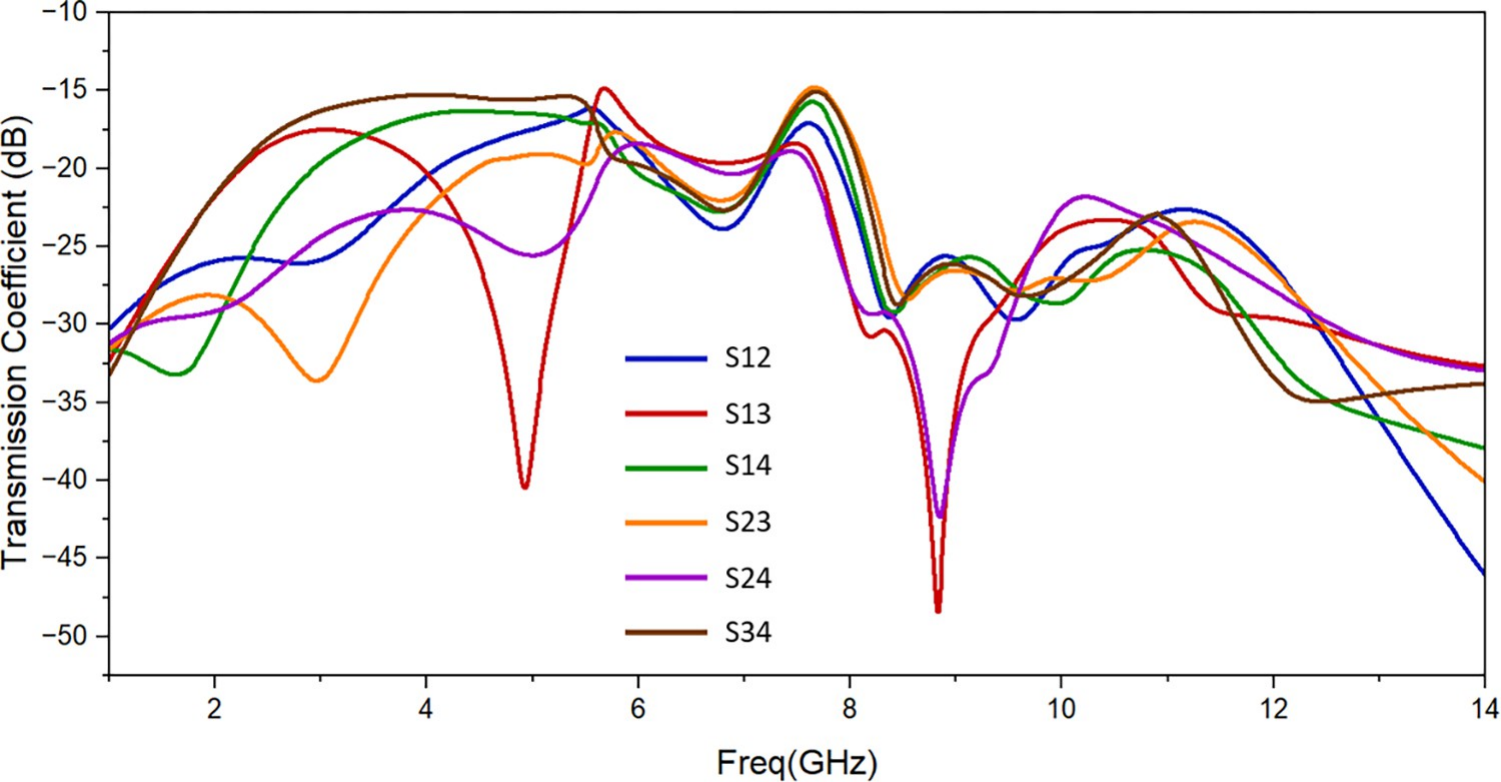
Proposed antenna’s simulated isolation.

Fig 7 displays the simulated reflection coefficient of designed antenna. It resonates from 2.57 GHz to 12.20 GHz with suitable values of return loss. Fig 8 shows the simulated findings, which provide less than -14 dB of isolation throughout the resonant frequency spectrum. The isolation attained complies with the standard MIMO antenna requirements.

## 3. Result analysis

**Fig 9:**
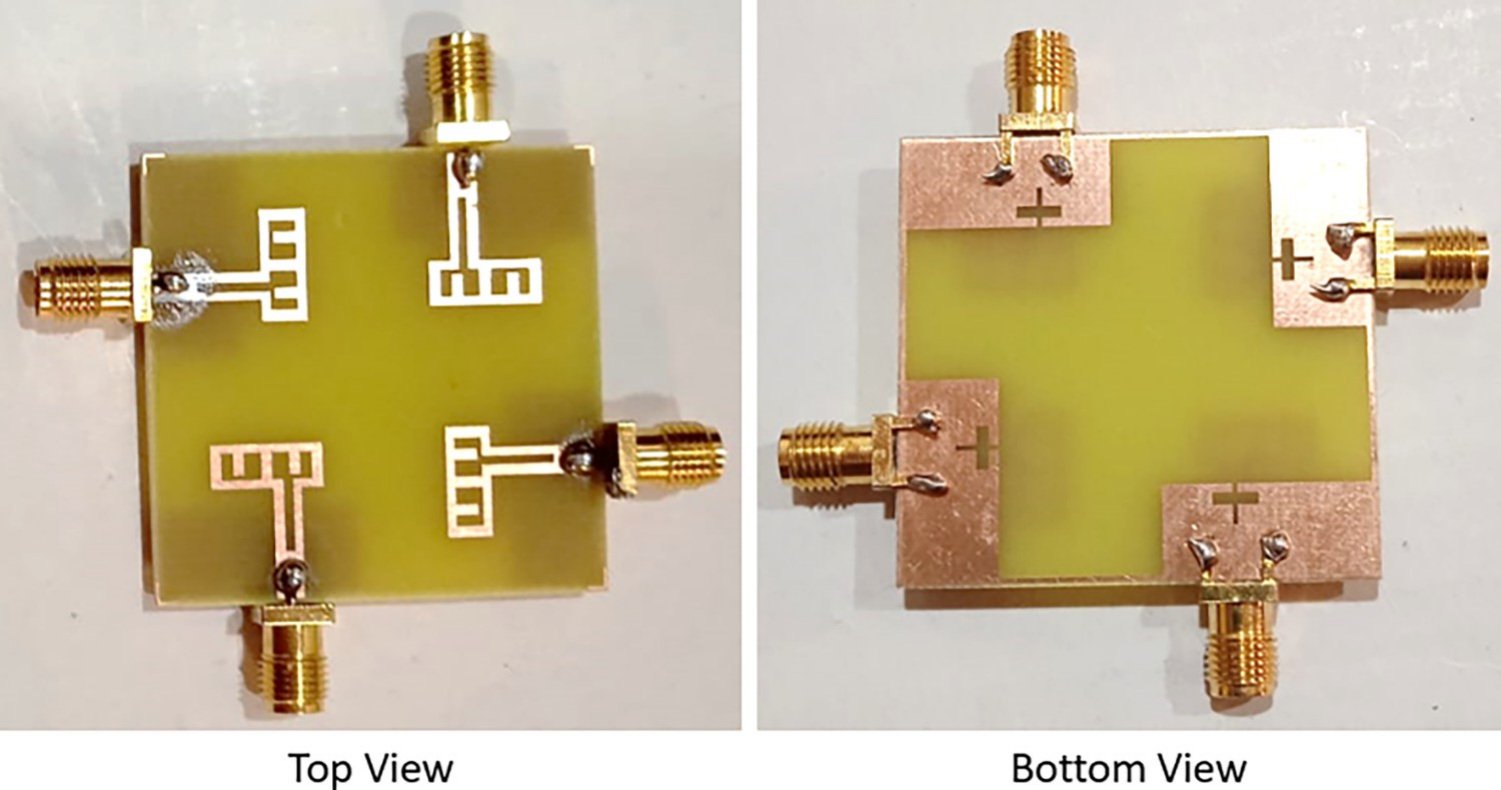
Suggested fabricated antenna.

The suggested antenna that’s been constructed and displayed in Fig 9 is measured using the Vector Network Analyzer. As demonstrated in Table 2, the simulation findings and experimental results are contrasted. It is shown that the findings from simulation and measurement nearly match one another. Errors in fabrication and soldering are the principal causes of the minor discrepancies between them.

**Table 2:** Results of the recommended antenna from simulations and measurements.

Result	Frequency band	Isolation (dB)
Simulated	2.57 GHz -12.20GHz	< -14 dB
Measured	2.68 GHz -12.22 GHz	< -13dB

**Fig 10:**
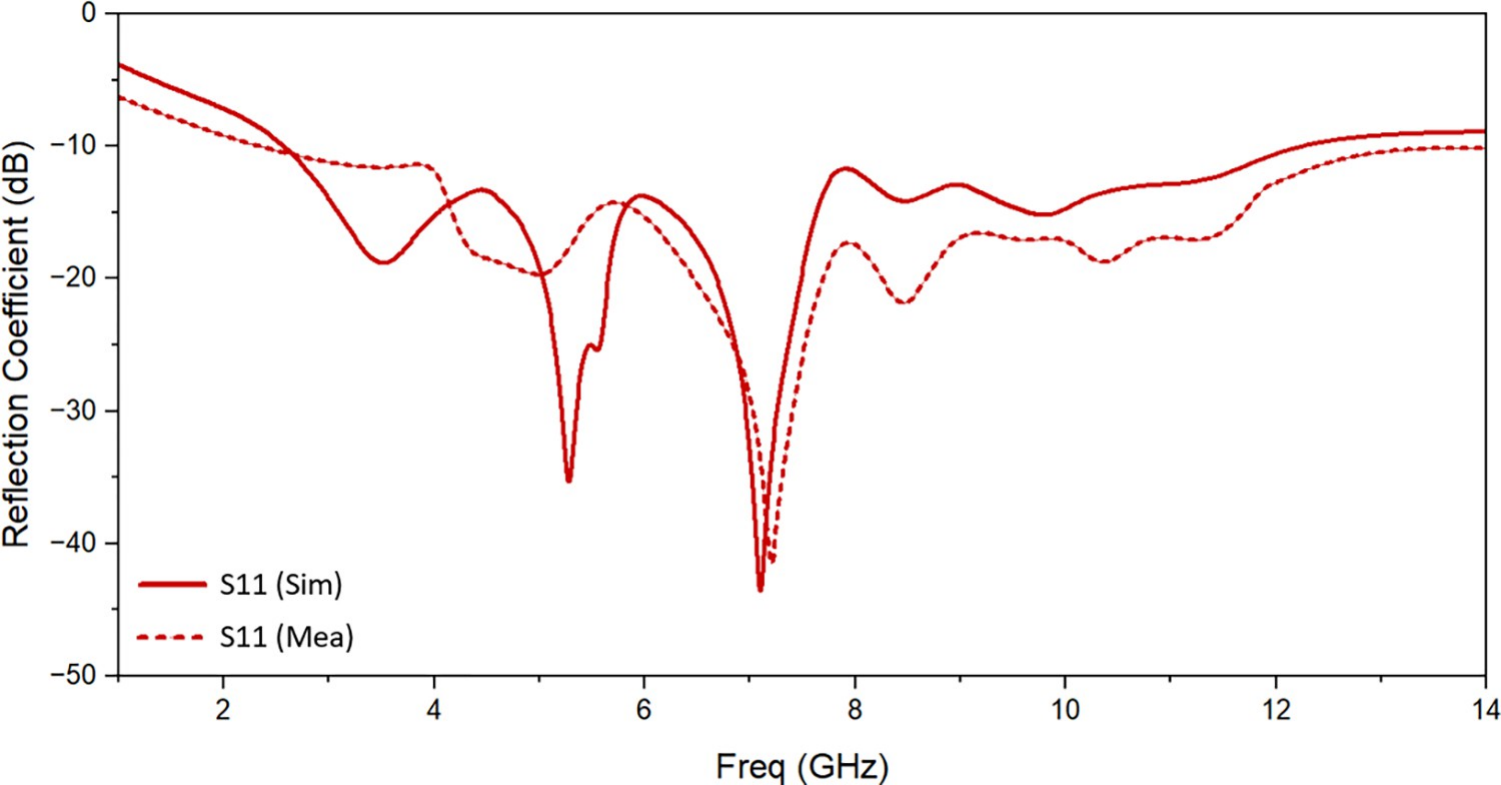
Reflection coefficient of the proposed antenna: Measured and simulated.

**Fig 11:**
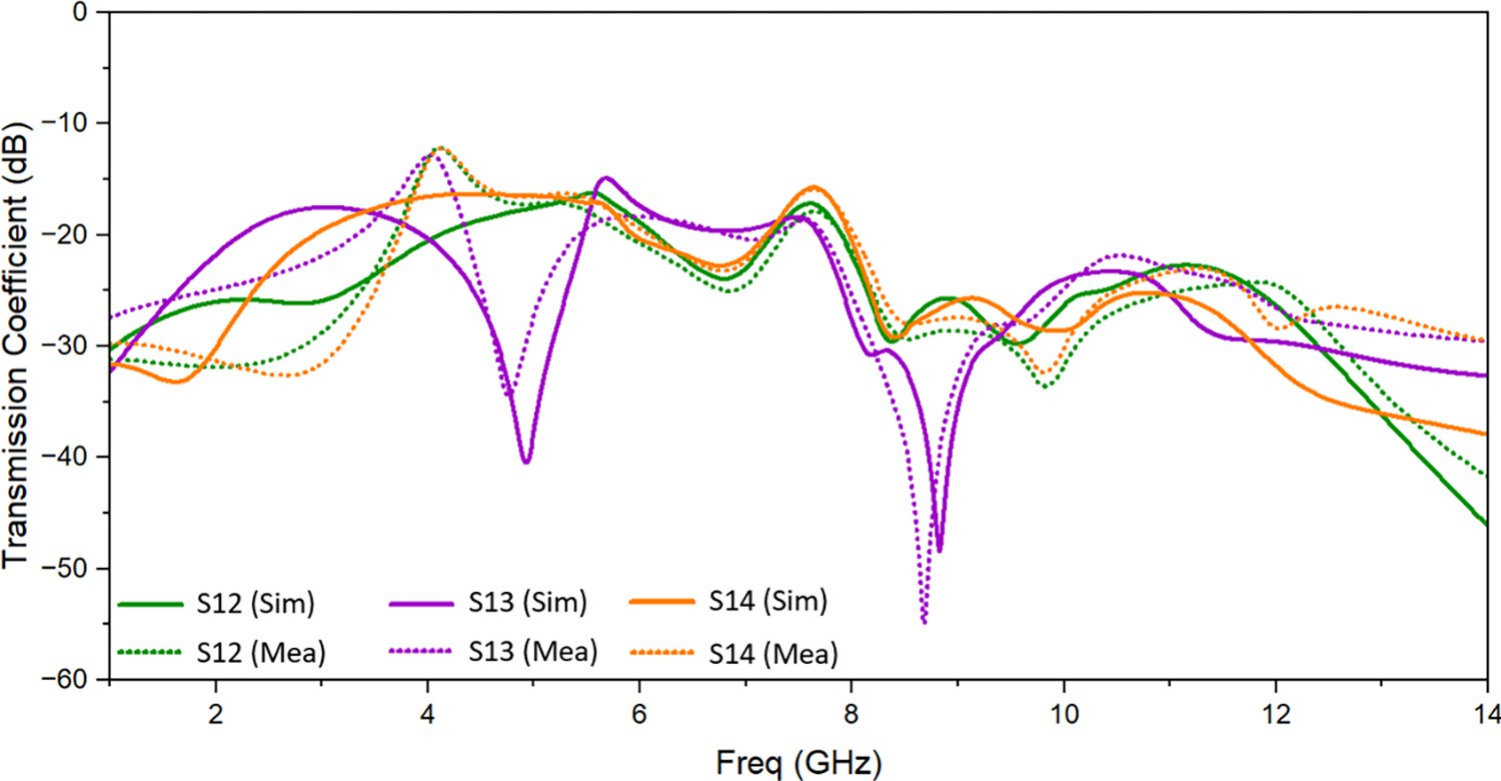
Transmission coefficient of the proposed antenna: Measured and simulated.

Simulated and observed reflection coefficients and transmission coefficients of the suggested UWB MIMO antenna are shown in Fig 10 and Fig 11 respectively. There is a good matching of simulated and observed reflection coefficient of port 1 as shown. It is evident that measured value of isolation between the resonating elements is of less than -13 dB throughout the resonating frequency band. One suggested antenna element (port 1) is energized during the measurement, and the remaining components are matched with the 50 Ω loads.

**Fig 12:**
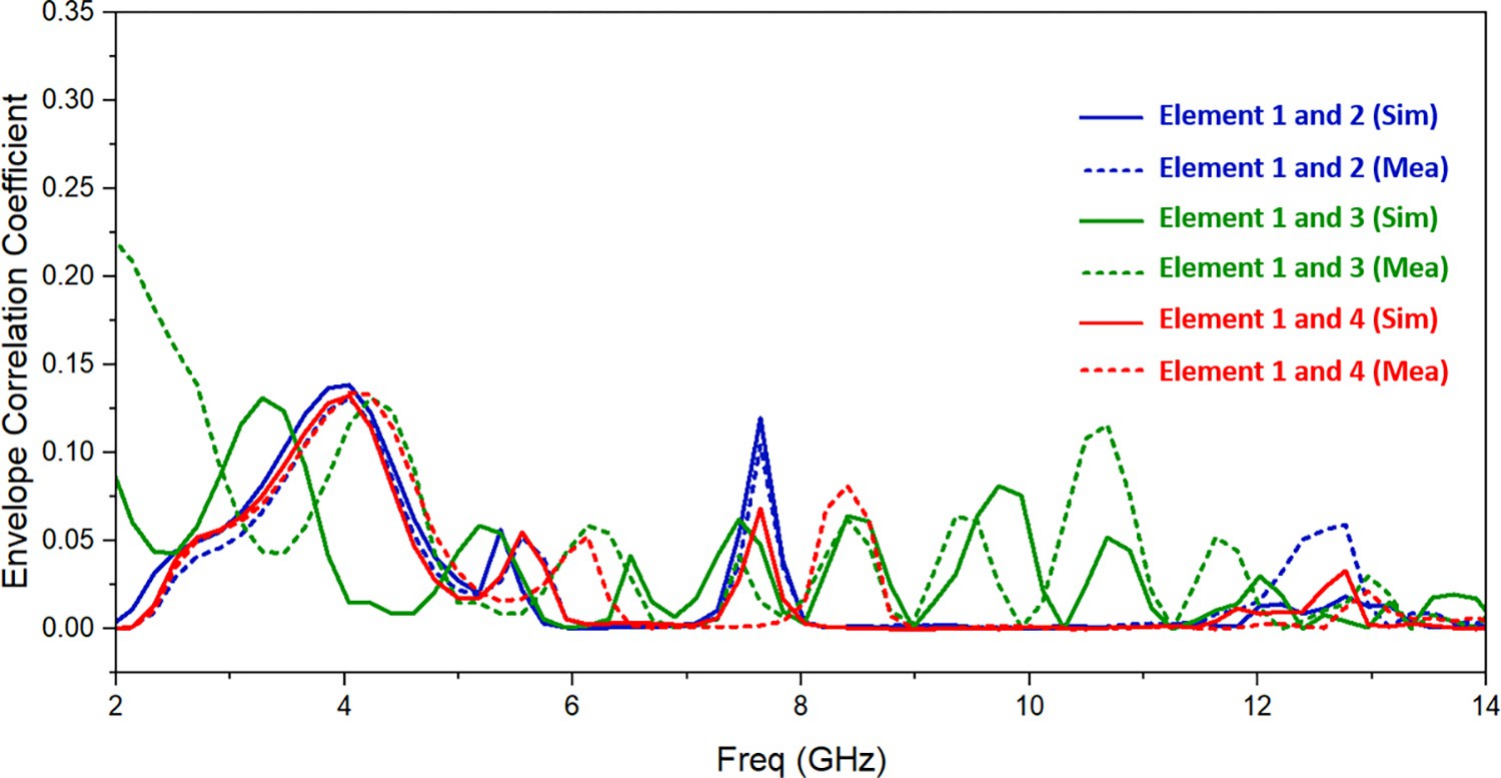
Simulated and observed ECC of suggested antenna.

The ECC is used to assess the planned antenna’s performance for diversity uses. It is used to assess how various RF signal pathways will influence how a signal gets to the receiver antenna. It can establish the correlation between several channels in a MIMO system. MIMO antenna should have ECC value between 0 and 1. The ECC for a multiple antenna system is written as [30]:

$$\rho e=ECC=\frac{|\iint [\vec{E1}(\theta ,\emptyset )\vec{E2}(\theta ,\emptyset )]d\Omega {|}^{2}}{\iint |\vec{E1}(\theta ,\emptyset ){|}^{2}d\Omega \iint |\vec{E2}(\theta ,\emptyset ){|}^{2}d\Omega }$$

where E is the pattern of the radiated field, Ω is solid angle, ϕ is azimuthal angle, and θ is elevation angle. ECC is calculated using 3D radiation patterns measured in an electromagnetic anechoic chamber. The diversity characterization of the UWB MIMO antenna design is shown in Fig 12, and it can be seen that ECC is less than 0.15 for entire resonating band.

Diversity gain (DG) indicates the transmission power loss when a multiple-input multiple-output system is used to implement the diversity mechanism. Diversity gain (DG) is the transmission power loss that occurs when a MIMO system applies the diversity mechanism. Equation may be used to derive DG, which is another important antenna feature [31].


$$DG=10\sqrt{1-{\left|ECC\right|}^{2}}$$

**Fig 13:**
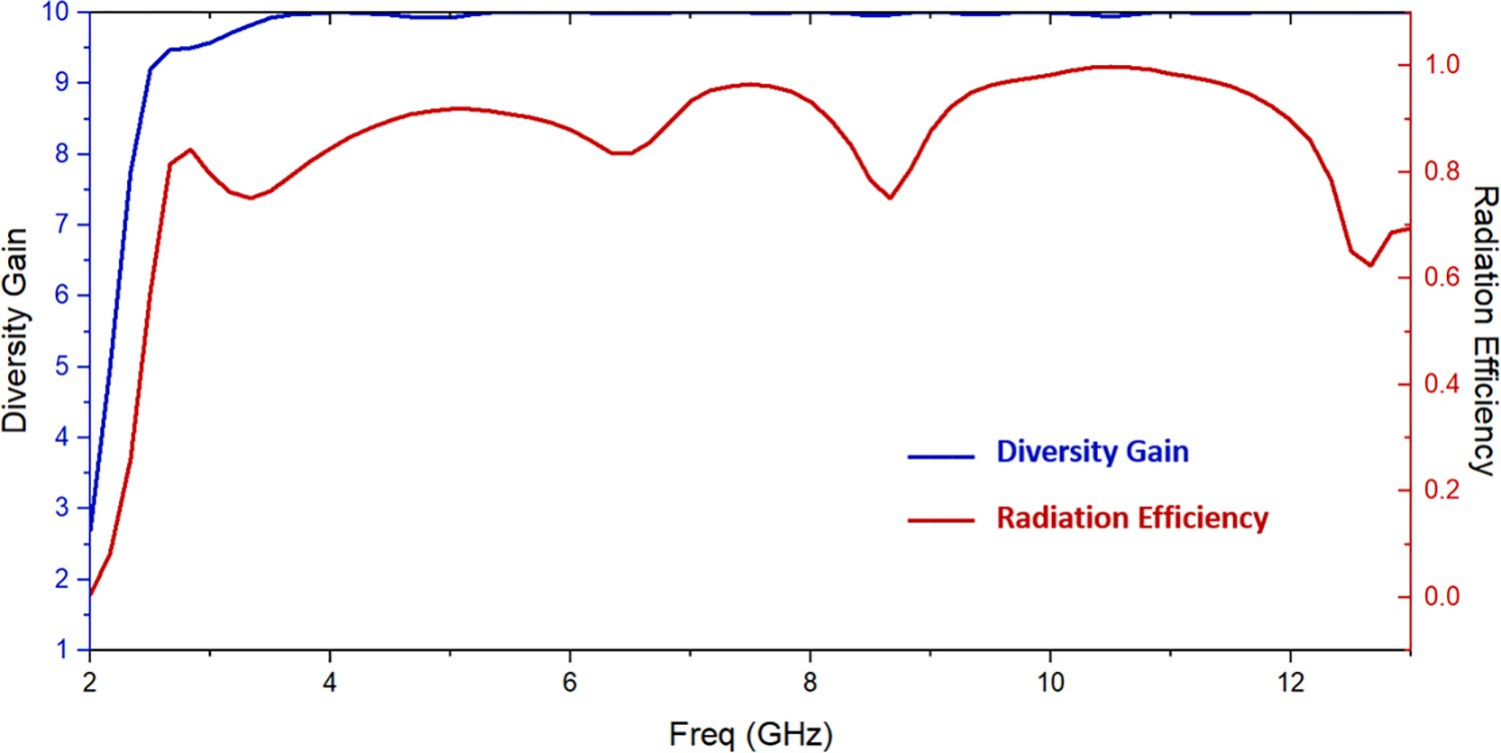
Diversity gain and radiation efficiency of suggested antenna.

The values of DG are greater than 9.5, as Fig 13 shows. To sum up, there is sufficient diversity in the suggested antenna.

Fig 13 displays the simulated radiation efficiency. For the whole UWB frequency range, it is greater than 74%. The antenna’s radiation efficiency is comparatively consistent.

**Fig 14:**
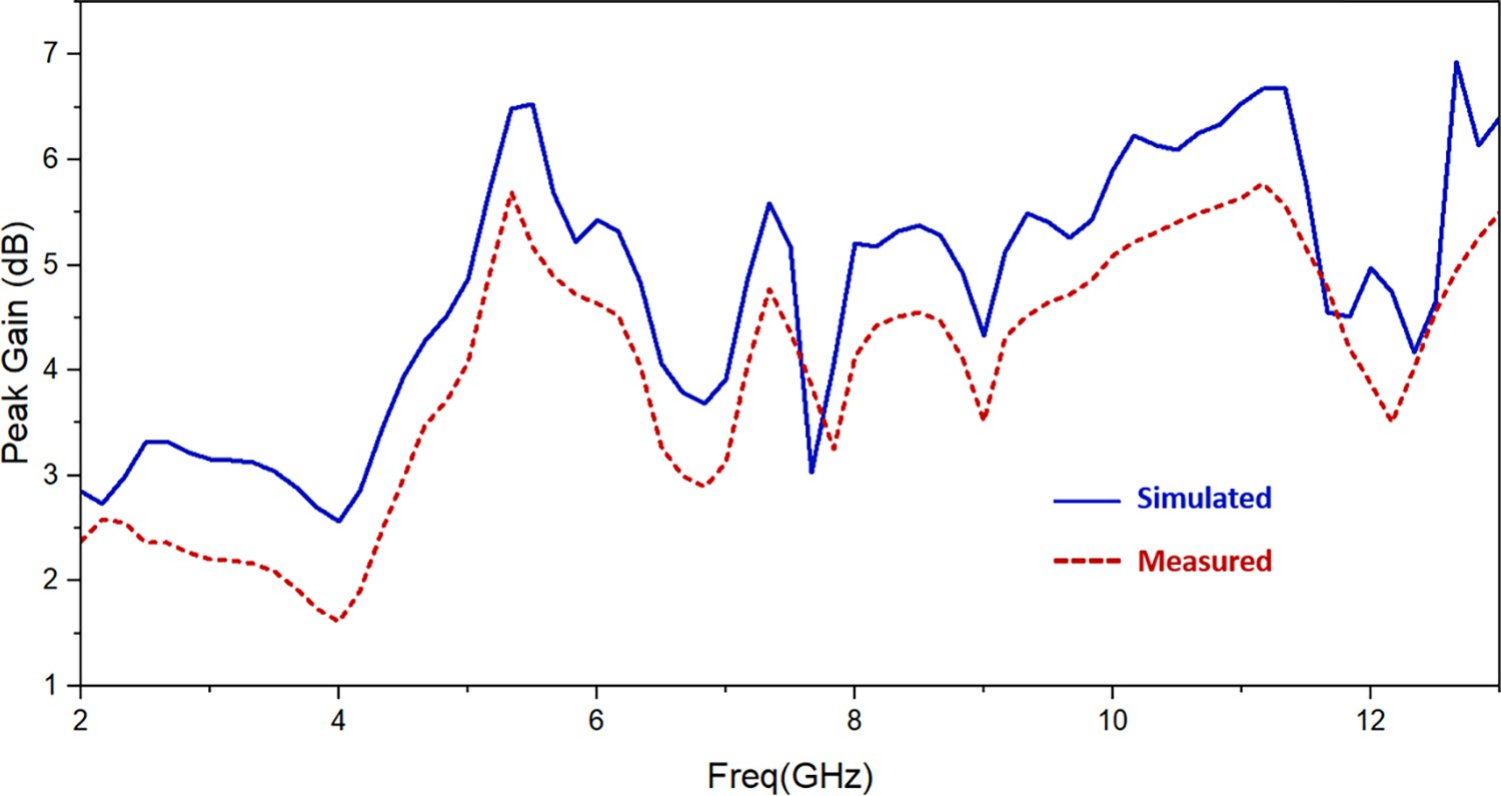
Simulated and observed gain of suggested antenna.

Fig 14 displays the simulated gain as well as the observed gain. The gain is dependent on directivity at these frequencies, hence despite having the efficiency factor consistent the gain varies across the UWB spectrum. The gain value spans from 2.5 dBi to 6.8 dBi between 2.57 GHz and 12.20 GHz, with an average gain of 4.7 dBi. A few differences exist between the measured and simulated gain. The reason is that three 50 Ω matched loads and the SMA connectors soldered at the antenna’s ports act as metal conductors, which reflect electromagnetic waves. The radiation and gain of the antenna will therefore be impacted by these metal conductors.

**Fig 15:**
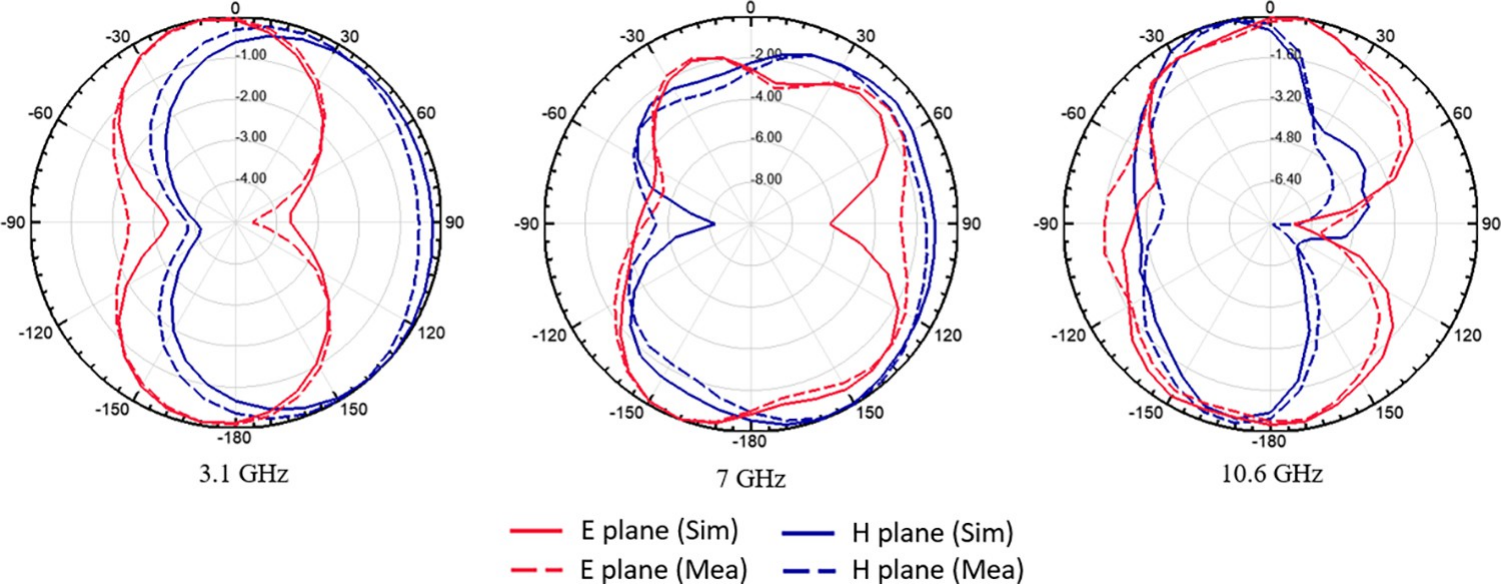
Simulated and measured normalized radiation pattern (dB).

The E-plane and H-plane’s 2D normalised simulated and observed radiation patterns at 3.1 GHz, 7 GHz, and 10.6 GHz are shown in Fig 15. The results demonstrate that there is considerable agreement between the simulated and bi-directionally observed radiation patterns.

The comparison between the UWB MIMO antenna under discussion and the previously described work is displayed in Table 3. It is evident that at resonance frequencies, the recommended antenna is small and has a suitable isolation and gain value. Compared to the other antennas shown, the recommended antenna is smaller. Additionally, the proposed antenna’s ground surface exhibits the same voltage level, which makes it appropriate for the intended uses [32–35].

**Table 3:** Comparison of designed UWB MIMO antenna with aforementioned work.

Ref	No. of Ports	Size (mm)	Relative Dimensions	Resonating Frequency (GHz)	Isolation (dB)	Peak gain (dB)	Connected Ground	Radiation Efficiency
[20]	2	47 × 93	0.49λ × 0.97λ	3.1~10.6	> 31	3.5	Yes	>70%
[21]	4	75.10 × 75.19	0.78λ × 0.78λ	3.1~17.3	> 13	5.5	Yes	Not Given
[31]	4	60 × 60	0.60λ × 0.60λ	3~11	> 20	3.4	No	>68%
[22]	4	58 × 58	0.58λ × 0.58λ	3~13.5	> 22	2.9	No	Not Given
[23]	4	60 × 60	0.54λ × 0.54λ	2.7~10.6	> 15	3.5	No	Not Given
[24]	4	45 × 45	0.46λ × 0.46λ	3.1~11	> 16	4.3	Yes	Not Given
[25]	4	45 × 45	0.46λ × 0.46λ	3.1~13.1	> 17	4.0	Yes	>73%
[26]	4	50× 39.8	0.42λ × 0.33λ	2.5~12	> 17	Not Given	No	Not Given
[27]	4	42 × 42	0.42λ × 0.42λ	3~11	> 15	3.5	No	>70%
[28]	4	38.3 × 38.3	0.38λ × 0.38λ	3~13.2	> 17	4.1	Yes	>72%
This Work	4	40 × 40	0.34λ × 0.34λ	2.57~12.2	> 14	4.7	Yes	>74%

## 4. Conclusion

This study describes a compact, four-port UWB MIMO antenna that is 40 mm × 40 mm × 1.6 mm overall. The suggested antenna has a good isolation and can operate in the entire UWB band (2.57–12.20 GHz). The predicted and observed radiation pattern, isolation, and return loss are in agreement. The antenna gain is 4.7 dBi on average. With an ECC of less than 0.15 and DG of around 10 dB, the suggested antenna has good diversity performance. To sum up, the suggested antenna shows great potential for wireless UWB-MIMO applications.
